# Individual Difference Factors in the Learning and Transfer of Patterning Discriminations

**DOI:** 10.3389/fpsyg.2017.01262

**Published:** 2017-07-28

**Authors:** Elisa Maes, Elias Vanderoost, Rudi D'Hooge, Jan De Houwer, Tom Beckers

**Affiliations:** ^1^Faculty of Psychology and Educational Sciences, Centre for the Psychology of Learning and Experimental Psychopathology KU Leuven, Leuven, Belgium; ^2^Laboratory for Biological Psychology, Faculty of Psychology and Educational Sciences KU Leuven, Leuven, Belgium; ^3^Learning and Implicit Processes Lab, Department of Experimental Clinical and Health Psychology, Ghent University Ghent, Belgium

**Keywords:** associative learning, patterning, rule-based generalization, feature-based generalization, visual processing style, mental representations

## Abstract

In an associative patterning task, some people seem to focus more on learning an overarching rule, whereas others seem to focus on acquiring specific relations between the stimuli and outcomes involved. Building on earlier work, we further investigated which cognitive factors are involved in feature- vs. rule-based learning and generalization. To this end, we measured participants' tendency to generalize according to the rule of opposites after training on negative and positive patterning problems (i.e., A+/B+/AB− and C−/D−/CD+), their tendency to attend to global aspects or local details of stimuli, their systemizing disposition and their score on the Raven intelligence test. Our results suggest that while intelligence might have some influence on patterning learning and generalization, visual processing style and systemizing disposition do not. We discuss our findings in the light of previous observations on patterning.

## Introduction

Qualitative differences exist between individuals in what they learn during associative learning tasks, even if their performance appears superficially similar. For example, in a concurrent associative pattering task, people are trained on negative and positive patterning problems simultaneously. In the negative patterning problems, stimuli (e.g., A and B) predict a certain outcome when presented alone, but not when presented in compound (A+/B+/AB−). In the positive patterning problems, presentation of a compound of two stimuli predicts the outcome, whereas presentation of the individual stimuli does not (e.g., C−/D−/CD+). To solve these simultaneous discrimination problems, people can either learn to associate each specific stimulus configuration presented to them with the absence or presence of the outcome or they can abstract the underlying rule of opposites (“compounds have the opposite outcome as the individual stimuli they are composed of”). Both strategies will result in the same response pattern. However, differences will emerge when participants are subsequently confronted with novel stimulus configurations. That is, when additionally trained on an incomplete pattern (e.g., E+/F+), they will respond in different ways to novel configurations derived from the incomplete training stimuli (e.g., EF). Participants who abstracted a rule of opposites during training can apply this rule to the novel configuration and may as a consequence predict no outcome on EF trials after E+/F+ training. In contrast, participants who simply learned to relate each stimulus configuration with its respective outcome without extracting an underlying rule can base their response to the novel configuration only on the degree of featural similarity between the novel configuration and the trained stimuli. Consequently, those participants will tend to predict the outcome on EF trials after E+/F+ training (Shanks and Darby, 1998). In previous studies, about half of participants exhibited rule-based rather than feature-based generalization after intermixed training on positive and negative patterning problems (Wills et al., 2011b; Maes et al., 2015; see further analysis reported in Wills, 2014).

The question is now which factors might correlate with those individual differences in performance during negative and positive patterning discrimination and generalization. Performance at the end of training seems to have an important influence on generalization strategy; participants achieving a high level of terminal performance during training showed a stronger tendency to respond in line with rule-based generalization during subsequent generalization testing (Shanks and Darby, 1998). Yet other results suggest that high training performance is not the only factor that determines generalization performance (Winman et al., 2005; Wills et al., 2011a,b; McDaniel et al., 2014; Little and McDaniel, 2015). In Experiment 2B of Maes et al. (2015), only half of the participants showed rule-based responding in a patterning task, despite the fact that all participants were trained to a performance criterion of at least 75%. Further, Wills et al. (2011b) observed that participants who received a working memory load during patterning training showed feature-based generalization despite reaching high levels of performance on the training items. Those results indicate that training accuracy is not the only determining factor in generalization style and that qualitatively different forms of learning might underlie similar levels of training performance; availability of working memory resources may be one important factor determining learning and subsequent generalization. Several other studies have also indicated that cognitive resources influence generalization strategy. For example, participants with a high working memory capacity displayed generalization consistent with the opposites rule, while participants with a low working memory displayed generalization consistent with featural overlap (Wills et al., 2011a). In a recent study of Cobos et al. (2017) individuals employed a feature-based generalization strategy when under strict time constraints, whereas individuals not under this strict time constrain demonstrated rule-based generalization. Further, results from the function-learning and categorization literature suggest that fluid intelligence might be another factor that determines generalization style (McDaniel et al., 2014; Little and McDaniel, 2015). However, in the category learning tasks involved, participants needed to pay attention to multiple dimensional features (e.g., color and shape) of a stimulus at the same time in order to classify stimuli correctly. It could be argued that this might have increased the role of intelligence in performance. The same argument can be made about the function learning tasks, because participants needed to map continuous inputs to continuous outputs. Such issues are not at play in patterning tasks that use dichotomous outcomes and involve stimuli that are not dimensional (e.g., food items). As such, the role of intelligence in determining generalization style might be overestimated from the experiments described above. Still, involvement of intelligence in generalization of patterning might be expected, based on at least three observations: (1) A correlation has been reported between the performance in concurrent negative and positive patterning and scores on the Raven's Advanced Progressive Matrices (Winman et al., 2005). From the report by Winman et al. (2005), however, it is unclear whether the patterning performance refers to performance during training, performance on generalization test trials or to rule awareness. (2) As mentioned above, working memory capacity has been shown to modulate feature- vs. rule-based generalization, as do time constraints (Wills et al., 2011a,b; Cobos et al., 2017). Working memory capacity and general intelligence are correlated (for a review, see Conway et al., 2003). (3) It has been found that rule-based generalizers had greater middle frontal cortex activity than feature-based generalizers in a patterning task (Milton et al., 2017), which might suggest stronger activation of higher cognitive functions in participants demonstrating rule-based generalization in a concurrent patterning task (Fuster, 2001; Siddiqui et al., 2008). A first goal of the research reported here was therefore to investigate whether the inclination toward feature- or rule-based generalization after concurrent negative and positive patterning training would indeed be associated with intelligence.

Other factors might also be at play during patterning tasks. Byrom (2013) has argued that negative patterning discrimination requires learning about a configuration independently from learning about its constituent stimuli. Therefore, it was hypothesized that a visual tendency to perceive groups of stimuli as a unitary configuration rather than a cluster of co-occurring stimuli should improve negative patterning performance. As previous work showed that individuals differ in their tendency to focus on global information vs. local details (Navon, 1977), it was argued that this variation might be related to variation in learning non-linear discriminations. In line with this idea, Byrom and Murphy (2014) found that people who have a tendency to focus on global aspects rather than local stimulus details (as measured with a Navon task, see below) discriminated better between BC and ABC in a modified negative patterning task (A+/BC+/ABC−). Based on this finding, in the current experiment, we investigated whether people with a more global processing style are also better in discriminating negative and positive patterning problems than people with a more local processing style. Importantly, the advantage of configural processing over elemental processing should if anything be stronger for simultaneous negative and positive patterning than for negative patterning alone. Exploratorily, we also assessed whether visual processing style was related to individual differences in feature- vs. rule-based generalization.

Finally, for exploratory reasons, we investigated whether systemizing is associated with rule-based generalization. Systemizing is the drive to analyze a system and derive the underlying rules that govern the behavior of the system (Baron-Cohen, 2002). The term system is used in a very broad sense here, and includes technical systems (e.g., a computer, a musical instrument), natural systems (e.g., the weather), and abstract systems (e.g., mathematics, syntax). Systemizing is thought to be an inductive processes that starts with data gathering and results in a rule about how the system works (Baron-Cohen, 2002; Baron-Cohen and Wheelwright, 2003). Therefore, a correlation between systemizing and generalization strategy might be expected.

In the current experiment, participants were first trained on complete and incomplete negative and positive patterning problems simultaneously (see Table 1). Thereafter, they were tested for generalization. Participants then performed a Navon task (to measure global vs. local visual processing style), followed by the Systemizing Quotient-Revised (SQ-R) questionnaire to measure systemizing (Wheelwright et al., 2006). Finally, they completed a computerized version of the Raven Standard Progressive Matrices test (RSPM; Raven, 1958) to measure non-verbal intelligence.

**Table 1:** Training and test trials for the patterning task; A–P represent different flavors of beverages; + indicates that drinking the beverage results in a happy mood; − indicates that drinking the beverage results in a sad mood.

**Training**			**Test**		
A+	B+	AB−	A	B	AB
C−	D−	CD+	C	D	CD
E+	F+	EF−	E	F	EF
G−	H−	GH+	G	H	GH
I+	J+		I	J	**IJ**
		KL−	**K**	**L**	KL
M−	N−		M	N	**MN**
		OP+	**O**	**P**	OP

*Trials in bold are the crucial generalization test trials*.

## Methods

### Participants and apparatus

Participants were 60 healthy volunteers (15 male, M_age_ = 21 years) who received either 12 euros or course credits for an undergraduate psychology course for their participation. This sample size ensured an *a-priori* power of 0. 82 to obtain a similar effect in size as the correlation between visual processing style and patterning discrimination reported by Byrom and Murphy (*R*^2^ = 0.13) at α = 0.05 (the size of the other potential effects of interest is difficult to gage from existing research, given the very different tasks used there). All tasks were presented on a computer running Affect software (Spruyt et al., 2010).

### Stimuli and materials

#### Patterning task

For half of the participants, Stimuli A–P were pictures of fruits, respectively, pineapple, cherry, strawberry, apple, banana, kiwi, raspberry, orange, passion fruit, grape, prune, gooseberry, melon, blackberry, lemon, and mango depicted in a can. For the remaining participants, the foods assigned to A and B were swapped with those assigned to C and D, and similarly for the other sets. Participants could respond by clicking on the “happy” or “sad” button presented on the screen. Further, participants could see the total number of points they gained.

#### Navon task

**Figure 1:**
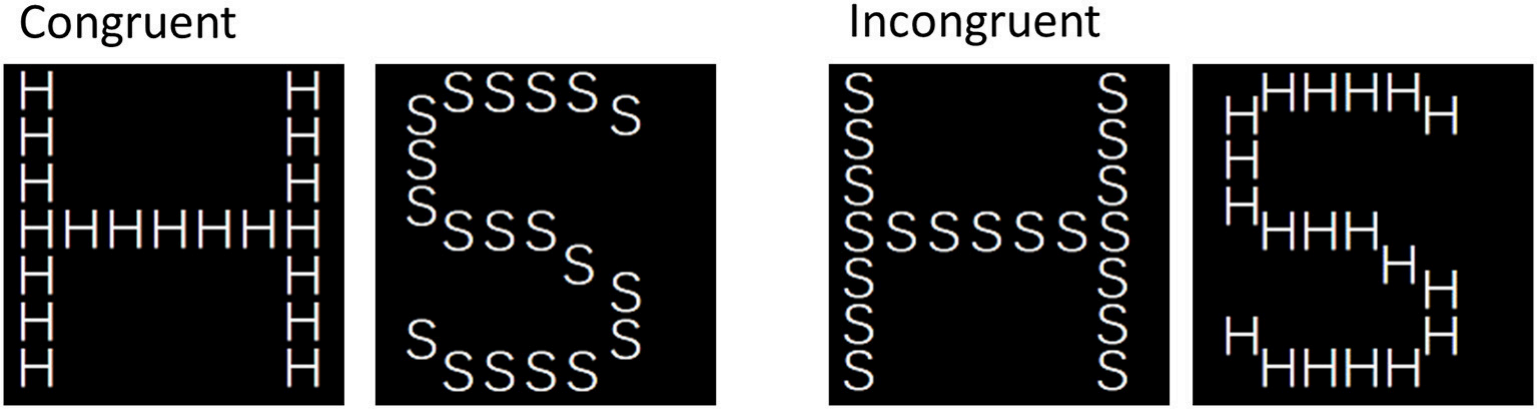
Stimuli used in the Navon task.

The stimuli used in this task closely resembled the stimuli used by Byrom and Murphy (2014). All stimuli consisted of large letters (S or H) composed of smaller letters (S or H), yielding four different stimuli (see Figure 1). Stimuli were presented in a black square spanning 55 × 55 mm (12.6° × 12.6°), with the small letters spanning approximately 5 × 5 mm (1.2° × 1.2°).

#### SQ-R questionnaire

A purpose-made Dutch translation of the SQ-R questionnaire (Wheelwright et al., 2006) was used to measure systemizing. The SQ-R consists of 75 questions (e.g., “I find it easy to use train timetables, even if this involves several connections,” “When I learn a language, I become intrigued by its grammatical rules.”) answered on a 4-point scale ranging from “strongly agree” to “strongly disagree” and scored from 0 to 2. Total scores on the test range from 0 (low interest in systemizing) to 150 (maximum score, extremely high systemizing).

#### RSPM

A computerized version (Mindsware, Geldermalsen, Netherlands) of the Raven Standard Progressive Matrices (RSPM) test (Raven, 1958) was used to measure intelligence.

### Procedure

All participants gave written informed consent for their participation and the procedure was approved by the social and societal ethics committee of the KU Leuven. All experiments were embedded in a cover story describing a pharmaceutical company that developed new kinds of beverages that could change the mood of a consumer to “happy” or “sad.”

#### Patterning task

**Figure 2:**
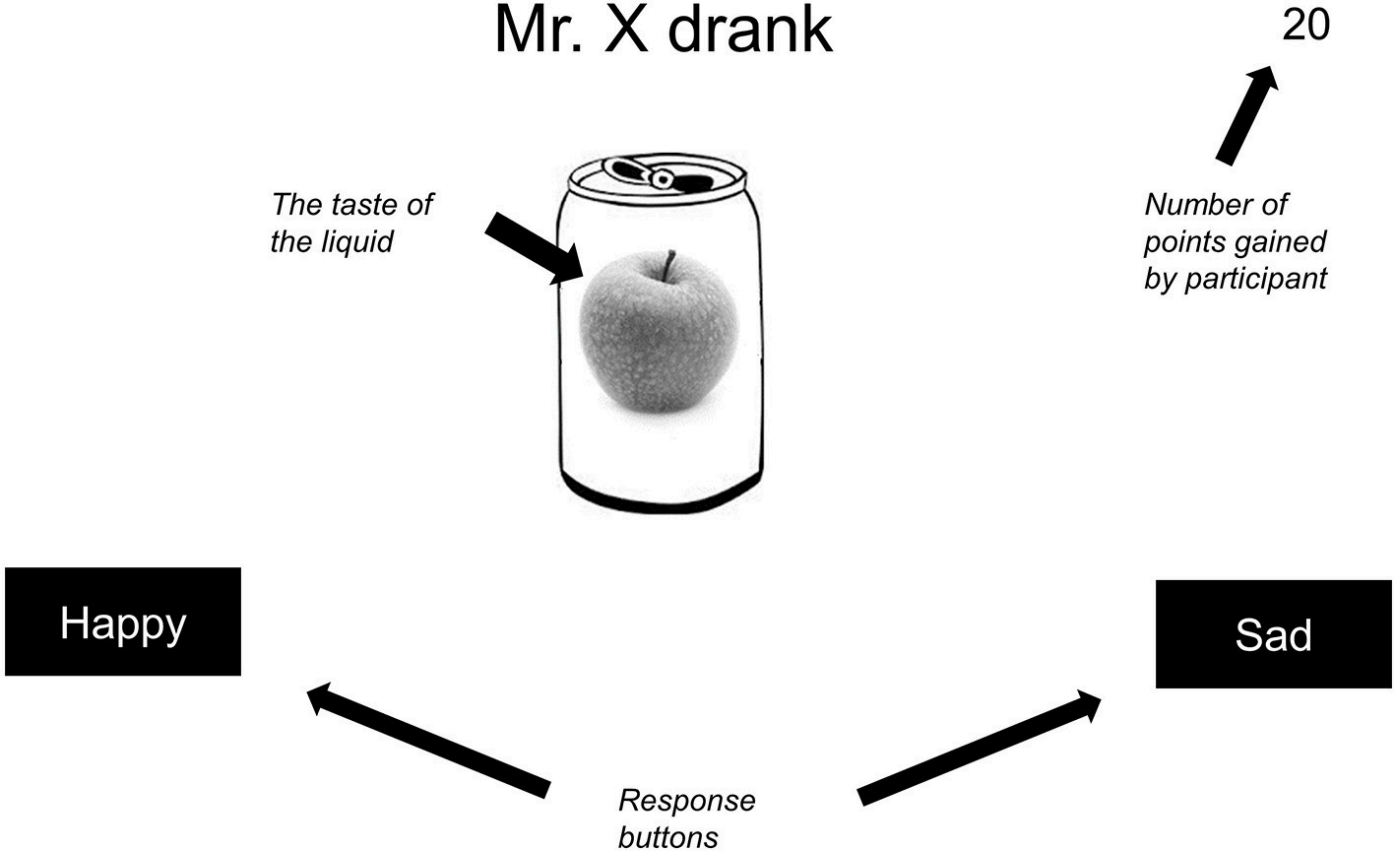
Stimuli as presented on the computer screen during a patterning trial (translated to English). Information in italics is added. Note that colors were removed and that the stimulus dimensions are increased to improve the readability, and are not proportional to the actual dimensions used.

The participants were informed that they needed to learn which beverages (indicated by different tastes of fruit) would lead to a happy mood after consumption and which ones would lead to a sad mood; they would gain 20 points for each correct answer (see Figure 2 for a visual representation of the stimulus display in a single training trial). Participants received up to 10 blocks of training, each block comprising two presentations of the 18 training trial types shown in Table 1 in a random order. No time limit for responding was imposed. During training trials a feedback message was displayed during at least 1,500 ms, after which participants could press enter to move on to the next trial. Participants moved on to the test phase when they reached a criterion of at least 32 correct responses in a given 36-trial block (89% correct). The transition to the test phase was accompanied by instructions that feedback would no longer be provided, but that the computer would still keep track of participants' scores. The test phase consisted of two blocks, each comprising one presentation of the 24 test trial types shown in Table 1, in a random order.

#### Navon task

The procedure used in this task resembled the procedure used by Byrom and Murphy (2014) as closely as possible. Participants were informed that they would be presented with large letters (S or H) composed out of smaller letters (S or H) and that, in successive trial blocks, they would have to identify either the large (global block) or the small letter (local block) by pressing the S or H key as quickly as possible. Half of the participants started out identifying the large letter, whereas the other half started out identifying the small letter. Global and local blocks alternated until participants completed four blocks of each type. Each 16-trial block contained four trials of each stimulus type (congruent S, congruent H, incongruent S, and incongruent H). Each trial began with the presentation of a fixation cross for 500 ms, followed by the presentation of a single stimulus in the center of the screen. After 40 ms the stimulus disappeared and a masking stimulus was presented until the participant made a response. Trial order within each block was semi-random with no more than two consecutive trials of the same stimulus type.

After completing the patterning task and the Navon task, participants were asked to fill in the SQ-R. Finally, they completed the RSPM task. No time limit was set to complete the different parts of the experiment.

## Results

Data and analysis scripts are available at Open Science Framework (https://osf.io/48tc3). Statistical analyses were conducted using both frequentist statistical techniques and Bayesian hypothesis testing. A Bayes Factor (BF) quantifies the strength of the relative statistical evidence for two rivaling hypotheses. It expresses the relative probability of the data under, e.g., the null vs. the alternative hypothesis (Gallistel, 2009; Rouder et al., 2009; Dienes, 2011; Morey, 2015). If a BF of about 1 is obtained, there is no evidence in favor of either one of the hypotheses, BFs above three can be regarded to provide substantial evidence in favor of the hypothesis that is in the numerator (or, conversely, values below one-third provide substantial evidence for the hypothesis in the denominator). We calculated BFs using JAPS 0.8.1.2 (JASP Team, 2017) and assuming a default prior distribution (Ly et al., 2016).

### Patterning task

None of the participants failed to meet the training criterion within the 10 blocks available; on average participants took 3.95 blocks (*SD* = 1.72). We further aimed at characterizing the learning process in more detail. More precisely, we were interested in investigating whether people tended to learn patterning problems in a stimulus-by-stimulus manner or an “once-for-all” manner. If the process of acquiring correct responding is a stimulus-by-stimulus process, performance is expected to increase gradually. In contrast, if the process is a one-for-all process, a sudden jump in performance is to be expected. This jump might indicate that the participant suddenly applied the rule of opposites, because rule application will boost performance (cfr. Mutter et al., 2007). In order to exploratorily investigate the acquisition pattern, we analyzed the difference in percentage correct responses between the penultimate (*M* = 76.57, *SD* = 9.17) and last training block (*M* = 93.24, *SD* = 3.17). A paired *t*-test confirmed that this differences was significant, *t*_(59)_ = −13.94, *p* < 0.01, 95% *CI* [−19.06, −14.27], *d* = −1.80, *BF*_*10*_ > 100. Although further research is required to confirm this exploratory result, it suggests that acquiring the correct responses in patterning learning may be an “once-for-all” process.

**Figure 3:**
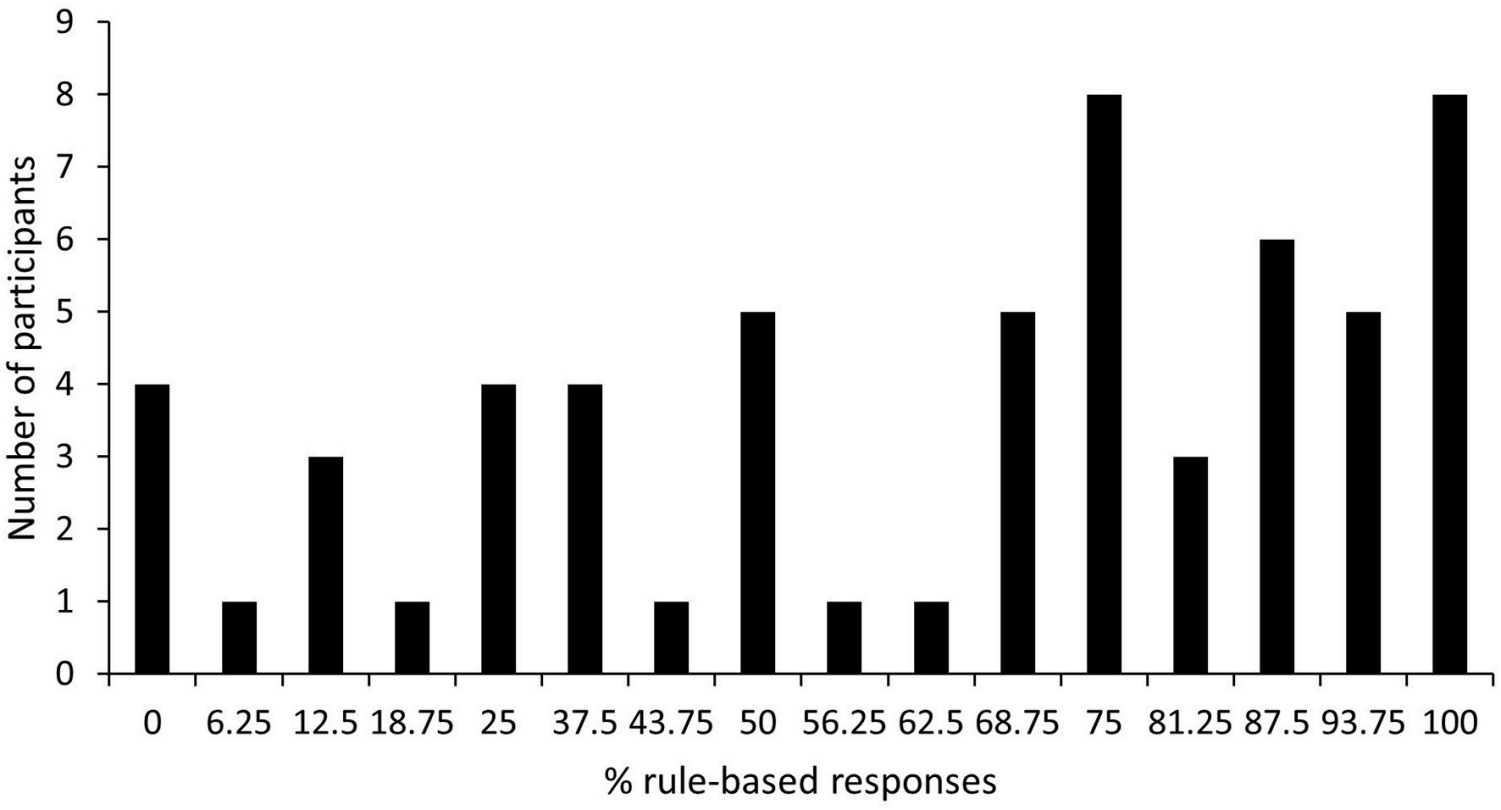
Distribution of percentage of rule-based generalization responses.

For each participant, the percentage of rule-based responses on the critical generalization trials was calculated as the sum of the number of rule-based responses to the elements (K, L, O, P) divided by eight (the number of element presentations) and the number of rule based responses to the compounds (IJ, MN) divided by four (the number of compound presentations). This sum was divided by 2 and multiplied by 100, such that 100% indicates rule-based responding on all trials and 0% indicates feature-based responding on all trials. At group level, participants exhibited a rule-based generalization strategy (*M* = 61.77%, *SD* = 31.85%), one-sample *t*-test comparing mean with 50%: *t*_(59)_ = 2.86, *p* < 0.01, 95% *CI* [53.54, 70.00], *d* = 0.37, *BF*_*10*_ = 5.62. It is clear from Figure 3, which depicts the distribution of percentage of rule-based responses, that generalization strategy was not simply bimodal (i.e., participants did not apply either a completely feature-based or a completely rule-based strategy). Rather a lot of variability exists in the percentage of rule-based generalization responses among participants with only 6.67% employing a complete feature-based strategy and 13.33% employing a complete rule-based strategy. Thirty-seven participants could be categorized as rule-based generalizers (>50% rule-based responses during generalization test), while 23 participants could be categorized as non-rule-based generalizers (≤50% rule-based responses during generalization test).

**Figure 4:**
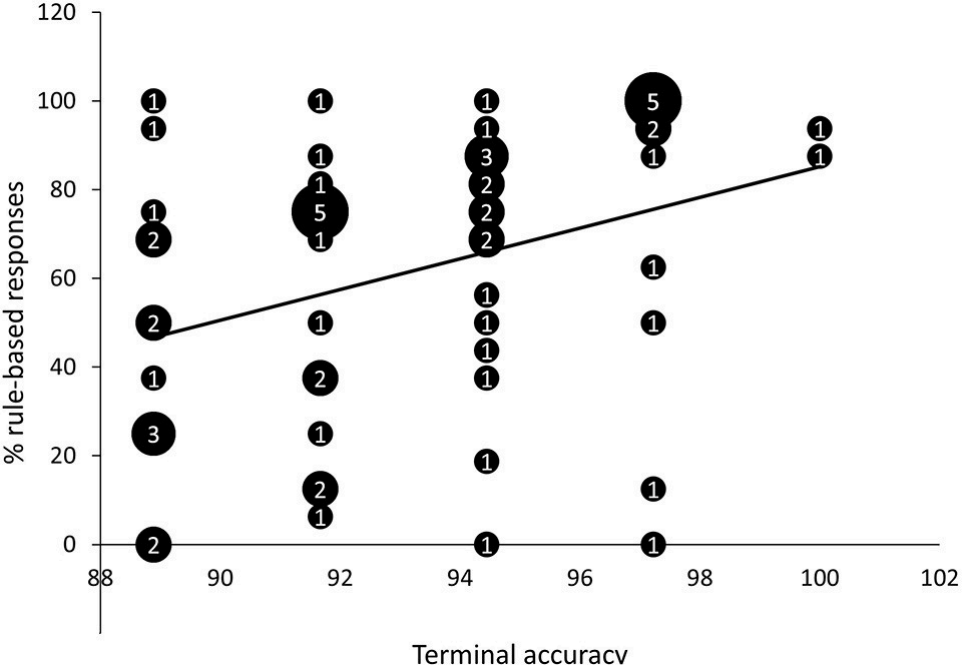
Scatter plot of percentage of rule-based generalization responses as a function of terminal accuracy (percentage of correct responses in last training block). The numbers presented in the bubbles reflect the number of participants with those particular scores.

We observed a positive correlation between terminal accuracy (i.e., the percentage of correct responses in the last training block; *M* = 93.24%, *SD* = 3.17%) and percentage of rule-based responses on generalization test trials, *r* = 0.34, *p* < 0.01, 95% *CI* [0.10, 0.55], *BF*_*10*_ = 5.53 (Figure 4). Learning speed (i.e., the number of training blocks needed to reach criterion; *M* = 3.95; *SD* = 1.72) and percentage of rule-based generalization responses were not significantly correlated, *r* = −0.16, *p* = 0.22, 95% *CI* [−0.40, 0.10], *BF*_*10*_ = 0.34.

**Figure 5:**
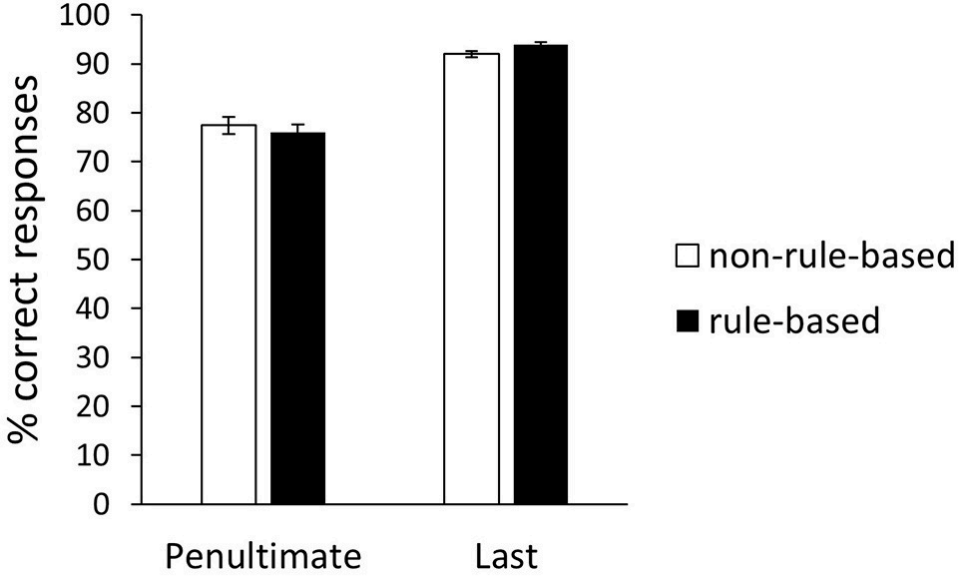
Percentage of correct training responses on penultimate and last training block for rule-based and non-rule-based generalizers. Error bars represent standard error of the mean.

It is conceivable that rule-based and non-rule-based generalizers differ in their acquisition pattern with rule-based generalizers showing a more “once-for-all” pattern and non-rule-based generalizers a stimulus-by-stimulus pattern. In order to investigate this, a repeated-measurements ANOVA with block as within-subjects variable and generalization type as between-subjects variable was conducted (Figure 5). An effect of Block was observed, *F*_(1, 58)_ = 177.78, *p* < 0.01, $\eta_{\text{partial}}^{2}$ = 0.75. Neither an effect of Generalization type, *F*_(1, 58)_ = 0.05, *p* = 0.83, $\eta_{\text{partial}}^{2}$ < 0.01, nor an interaction effect, *F*_(1, 58)_ = 1.86, *p* < 0.18, $\eta_{\text{partial}}^{2}\;=$ 0.03, were observed. For the Bayesian analysis, we utilized the Bayesian model comparison technique for factorial designs as introduced by Rouder et al. (2017) and Wagenmakers et al. (2017). In this case, there are five models to consider: (1) the “Null model” that contains only the grand mean, (2) the “Generalization type” model that contains the effect of generalization type, (3) the “Block” model that contains the effect of block, (4) the “Generalization type + Block” model that contains both main effects, and (5) the “Generalization type + Block + Generalization type ^*^ Block” model that includes both main effects and the interaction. All models, except the model containing generalization type, receive overwhelming evidence in comparison to the Null model (Table 2). However, the evidence against the two main effects model compared to the “Block” model is roughly a factor 4 (*BF*_*10*_ of “Block” model divided by *BF*_*10*_ of two main effects model). Further, the evidence against including the interaction compared to “Block” model was roughly a factor 6 (*BF*_*10*_ of “Block” model compared with *BF*_*10*_ of interaction model). In conclusion, the Bayesian analysis is in line with the standard NHST analysis and further supports the absence of an effect of generalization type. Additional comparisons revealed that the difference between the penultimate and the last block was significant for both the rule-based generalizers, *t*_(36)_ = −11.38, *p* < 0.01, 95% *CI* [−21.4, −14.75], *d* = −1.87, *BF*_*10*_ > 100, and the non-rule-based generalizers, *t*_(22)_ = −8.24, *p* < 0.01, 95% *CI* [−18.29, −10.93], *d* = −1.77, *BF*_*10*_ > 100 (Figure 5).

**Table 2:** JASP output table for the Bayesian repeated-measurements ANOVA with block as within-subjects variable and generalization type as between-subjects variable.

**Models**	**BF_10_**
Null model (incl. subject)	1.00
Generalization strategy	0.22
Block	2.08 × 10^24^
Generalization strategy + Block	5.64 × 10^23^
Generalization strategy + Block + Generalization strategy ^*^ Block	3.40 × 10^23^

*All models include subject*.

### Navon task

Response times on the Navon task were recorded for four different trial types: global congruent, local congruent, global incongruent, and local incongruent, where global and local refer to the level the participants were asked to respond to. We copied the data processing procedure of Byrom and Murphy (2014), analyzing response times from correct trials only and removing response times deviating one or more standard deviations from the mean for that trial type^1^. A visual processing score was calculated for each participant by subtracting the mean reaction time for global incongruent trials from the mean reaction times for local incongruent trials, higher scores reflecting faster identification of targets on global trials (Byrom and Murphy, 2014). The average visual processing score (*M* = −116.52 ms; *SD* = 209.52 ms) was significantly below zero, *t*_(59)_ = −4.31, *p* < 0.01, 95% *CI* [−170.65, −62.40], *d* = −0.56, *BF*_*10*_ = 331.6, suggesting that participants in our sample showed a bias toward the local level. While it is often assumed that humans have a global processing advantage (Navon, 1977), this advantage has not always been observed (e. g., Hoffman, 1980; Billington et al., 2008) and various factors have been reported to influence this tendency (for an overview see Kimchi, 1992).

To estimate the reliability of the Navon task, we calculated a visual processing score based on the first half of global incongruent and local incongruent trials and a visual processing score based on the second half of global incongruent and local incongruent trials. A strong correlation was observed between those split-halve scores, *r* = 0.75, *p* < 0.01, 95% CI [0.62, 0.85], *BF*_*10*_ > 100, indicating that our Navon task had a good internal reliability.

### SQ-R and RSPM

The average score on the SQ-R was 46.63 (*SD* = 13.86), which is somewhat lower than the mean score in previous work [Wheelwright et al., 2006: *M* = 55.56, *t*_(59)_ = −4.99, *p* < 0.01, 95% *CI* [−43.05, 50.21], *d* = −0.64, *BF*_*10*_ > 100]. All participants answered “slightly disagree” or “strongly disagree” on question 43 of the SQ-R (“If there was a problem with the electrical wiring in my home, I'd be able to fix it myself”) which resulted in a score of 0 for all participants on this question. Excluding this question resulted in a Cronbach's alpha of 0.84.

The average score on the RSPM was 52.55 (*SD* = 5.19). To calculate the Cronbach's alpha of the RSPM 10 items had to be removed because all participants replied correctly to those items and the variance was thus 0. After removing those items a Cronbach's alpha of 0.83 was obtained.

### Correlations between tasks

**Figure 6:**
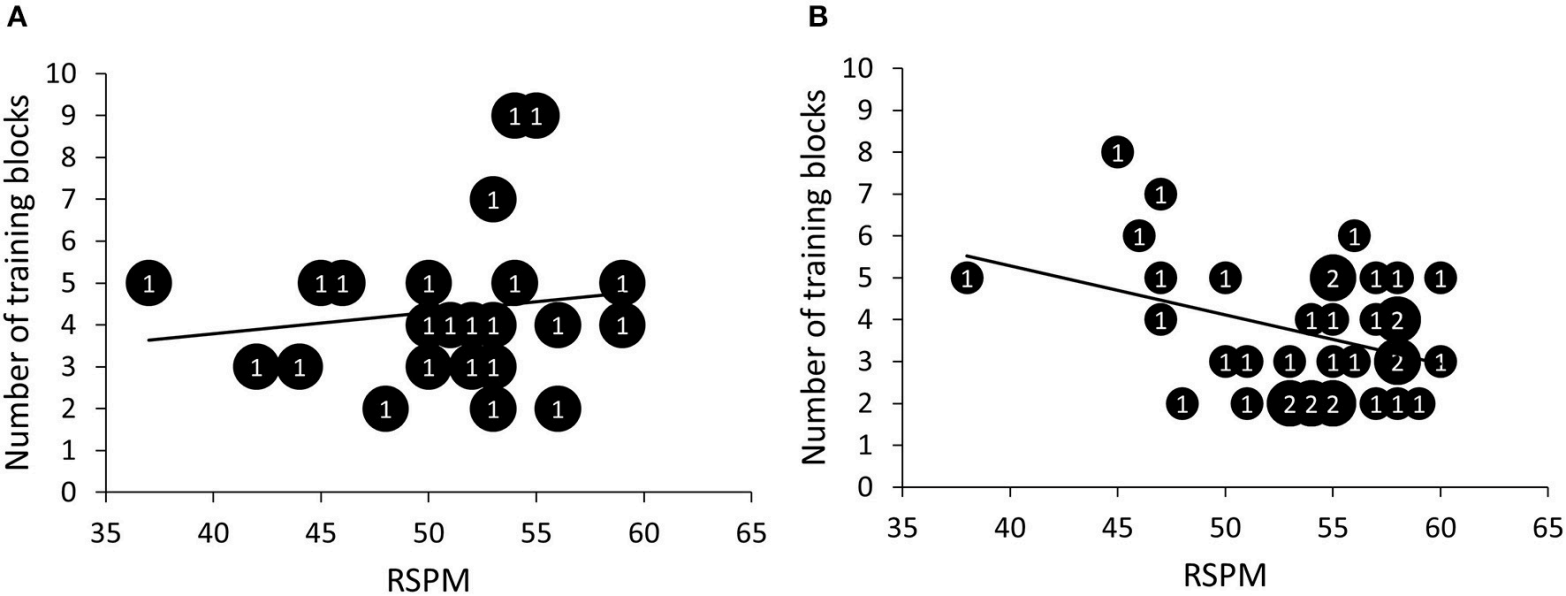
Scatter plots of number of training blocks needed to reach criterion as a function of scores on the Raven Standard Progressive Matrices (RSPM) test for non-rule-based generalizers **(A)** and for rule-based generalizers **(B)**. The numbers presented in the bubbles reflect the number of participants with those particular scores.

**Figure 7:**
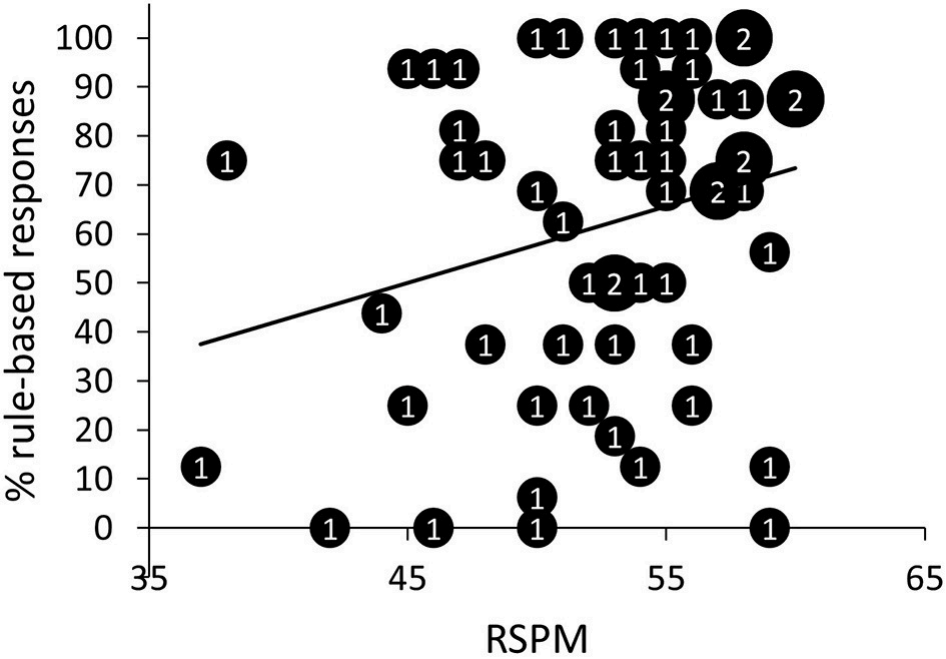
Scatter plot of the percentage of rule-based responses as a function of scores on the Raven Standard Progressive Matrices (RSPM) test. The numbers presented in the bubbles reflect the number of participants with those particular scores.

First, we investigated whether there was a relationship between RSPM and SQ-R on the one hand and patterning discrimination and generalization on the other hand. Three different parameters were used to assess patterning discrimination: learning speed or the number of training blocks needed to reach criterion (*M* = 3.95; *SD* = 1.72), overall accuracy during training (*M* = 76.12; *SD* = 3.81), and a discrimination difference score, calculated as the difference between the percentage of correct responses after the first block and the percentage of correct responses after the second block (*M* = 19.77; *SD* = 11.58; all participants took minimally two blocks to reach criterion)^2^. First, intelligence seemed not to correlate significantly with speed of learning, *r* = −0.18, *p* = 0.18, 95% *CI* [−0.41, 0.08], *BF*_*10*_ = 0.39. However, it is conceivable that intelligence is especially important for rule learning, but not so much for simply learning specific relationships. Therefore, we computed correlations separately for the rule-based and non-rule-based generalizers. For rule-based generalizers (>50% rule-based responses during generalization test), but not for non-rule-based generalizers, the number of training blocks needed to reach criterion decreased with increasing RSPM scores according to the standard null-hypothesis methodology, *r*_rule_ = −0.36, *p*_rule_ = 0.03, 95% *CI* [−0.62, −0.04], *BF*_*10*_ = 2.14; *r*_non-rule_ = 0.15, *p*_non-rule_ = 0.51, 95% *CI* [−0.28, 0.53], *BF*_*10*_ = 0.32^3^ (Figure 6). None of the other discrimination parameters correlated significantly with either RSPM or SQ-R. Table A1 in appendix provides detailed statistics of the correlations between the patterning discrimination parameters on the one hand and RSPM and SQ-R on the other hand. Further, participants with higher scores on the RSPM were more likely to show rule-based generalization, *r* = 0.25, *p* = 0.05, 95% *CI* [0.00, 0.48], although Bayesian analysis was indifferent between the presence or absence of a correlation, *BF*_*10*_ = 1.05 (Figure 7). SQ-R was not associated with rule-based generalization, *r* = 0.06, *p* = 0.67, 95% *CI* [−0.20, 0.31], *BF*_*10*_ = 0.18.

We next investigated whether we could replicate previous observations of a relationship between visual processing style and patterning discrimination, as observed by Byrom and Murphy (2014). Surprisingly, we failed to find a correlation between visual processing score and any of the patterning discrimination parameters. Bayesian analysis in fact provided evidence in support of the absence of a correlation for all three parameters (see Table 3 for detailed statistics). Byrom and Murphy reported a correlation between visual processing score and patterning discrimination with an *R*^2^ of 0.13, corresponding to a medium effect size (Cohen, 1992). Based on their power, our sample size yields an *a-priori* power of 0.82 to detect a comparable effect. Moreover, the standard error of the visual processing score (*SEM* = 27.05) was higher than the one reported by Byrom and Murphy (2014) (*SEM* = 1.20), providing ample room for a correlation with visual processing style to emerge. This suggests that our failure to find a correlation was not due to a lack of power. Visual processing style was not correlated with the percentage of rule-based generalization responses either (see Table 3).

**Table 3:** Correlations between visual processing score and patterning discrimination parameters.

		**Visual processing score**
Number of training blocks	*r*	0.03
	*p*	0.79
	95% *CI*	[−0.22, 0.29]
	*BF*_*10*_	0.17
Overall accuracy	*r*	0.15
	*p*	0.25
	95% *CI*	[−0.11, 0.39]
	*BF*_*10*_	0.31
Discrimination difference	*r*	0.07
score	*p*	0.57
	95% *CI*	[−0.18, 0.32]
	*BF*_*10*_	0.19
Percentage of rule-based	*r*	0.10
generalization responses	*p*	0.43
	95% *CI*	[−0.15, 0.35]
	*BF*_*10*_	0.22

### Multiple linear regression

In order to discern which factors most prominently predict rule-based generalization a multiple linear regression was conducted. Given that some previous work suggests an influence of training performance (Shanks and Darby, 1998), terminal discrimination accuracy was entered in the model first. Thereafter, RSPM score, SQ-R score and visual processing score were entered into the model simultaneously.

The models without and with RSPM, SQ-R, and visual processing score both predict the percentage of rule-based generalization responses to a statistically significant degree, $R_{1}^{2}$ = 0.12, *F*_1(1, 58)_ = 7.79, *p*_1_ < 0.01, $R_{2}^{2}$ = 0.17, *F*_2(4, 55)_ = 2.89, *p*_2_ = 0.03. The change in *R*^2^ which was not significant, $R_{change}^{2}$ = 0.06, *F*_(3, 55)_ = 1.23, *p* = 0.31, and only terminal accuracy seemed to contribute significantly to the prediction of generalization performance in either model (see Table 4). Bayesian analysis provides substantial evidence in favor of the alternative model over the null model for both the model that contains terminal accuracy only (*BF*_*10*_ = 6.18) and the model that contains terminal accuracy and RSPM score (*BF*_*10*_ = 4.67; see Table 5). We also calculated the Bayes factor for each model relative to the full model BF_mf_, which is given by BF_mf_ = BF_m0_/BF_f0_ (Rouder and Morey, 2012). The model with the greatest evidence relative to the full model is the model with only terminal accuracy as a predictor (see Table 5).

**Table 4:** Coefficients of the regression model.

**Model**		***b_1_***	***SE_b_***	***t***	***p***	**95% CI**
1	(Constant)	−260.75	115.65	−2.26	0.03	[−492.24, −29.27]
	Terminal accuracy	3.46	1.24	2.79	0.007	[0.98, 5.94]
2	(Constant)	−302.49	119.50	−2.53	0.01	[−541.97, −63.01]
	Terminal accuracy	3.13	1.28	2.45	0.02	[0.57, 5.69]
	RSPM score	1.19	0.78	1.54	0.13	[−0.36, 2.75]
	SQ-R score	0.25	0.29	0.88	0.38	[−0.32, 0.82]
	Visual processing score	0.02	0.02	0.87	0.39	[−0.02, 0.05]

*Model 1 included terminal accuracy as predictor. Model 2 additionally includes RSPM score, SQ-R score and visual processing score as predictors. SE_b_, Standard error of b_1_; RSPM, Raven Standard Progressive Matrices*.

**Table 5:** Results of Bayesian linear regression with terminal accuracy, RSPM score, SQ-R score, and visual processing score as predictors.

**Models**	**BF_10_**	**BF_mf_**
Null model	1.00	
Terminal accuracy	6.18	4.65
RSPM	1.37	1.03
Terminal accuracy + RSPM	4.70	3.53
SQ-R	0.28	0.21
Terminal accuracy + SQ-R	2.60	1.95
RSPM + SQ-R	0.53	0.40
Terminal accuracy + RSPM + SQ-R	2.38	1.79
Visual processing	0.34	0.26
Terminal accuracy + Visual processing	2.48	1.87
RSPM + Visual processing	0.67	0.50
Terminal accuracy + RSPM + Visual processing	2.37	1.78
SQ-R + Visual processing	0.14	0.10
Terminal accuracy + SQ-R + Visual processing	1.24	0.93
RSPM + SQR + Visual processing	0.31	0.23
Terminal accuracy + RSPM + SQ-R + Visual processing	1.33	1.00

*BF_10_, Bayes factor in favor of the model compared to the null model; BF_mf_, Bayes factor for each model relative to the full model; RSPM, Raven Standard Progressive Matrices*.

## Discussion

In accordance with earlier studies (Wills et al., 2011b; Maes et al., 2015; see further analysis reported in Wills, 2014), we observed that about half of the participants exhibited rule-based generalization whereas the other half exhibited feature-based generalization after simultaneous negative and positive patterning training. Notwithstanding that exploratory results indicate that the acquisition pattern for the initial training stimuli did not differ between rule-based and non-rule-based generalizers. Further, a statistically-significant correlation between intelligence and rule-based generalization was observed (using a standard null-hypothesis methodology). Most importantly, visual processing was not correlated with patterning discrimination or rule-based generalization.

To some extent, individual differences in generalization performance observed in the current experiment may be the simple consequence of differences in the extent to which the various relations presented during training have been learned; humans will exhibit more accurate generalization if they have acquired the relations from which to generalize more firmly. However, even under conditions of high terminal accuracy (at least 89% correct), considerable individual variability was observed, with about half of the participants not generalizing according to the rule of opposites. Moreover, the regression model that included terminal accuracy as a predictor was able to explain only 12% of the variance observed in percentage of rule-based generalization responses. Those results support the idea that although differences in initial patterning performance exist, those differences cannot fully account for the observed differences in generalization performance. The capacity to learn the rule of opposites and the tendency to apply this rule to new stimuli might further contribute to the variation in generalization responses. The absence of a difference in acquisition pattern between rule-based generalizers and non-rule-based generalizers suggests that the latter factor (differences in application tendency) plays a role in determining generalization performance, although future research should confirm this.

The question raised here is whether certain cognitive factors correlate with the interindividual variance in generalization style. The first factor taken into consideration was intelligence. A correlation between RSPM scores and speed of learning was observed in the rule-based group (although Bayesian statistics was inconclusive), suggesting that fluid intelligence determines how fast people pick up on patterning rules. However, the results regarding the relation between fluid intelligence and generalization are more equivocal. While the results of null hypothesis significance testing suggest a correlation between RSPM scores and the percentage of rule-based generalization responses, Bayesian analysis is indecisive. Further, RSPM scores did not seem to predict the percentage of rule-based generalization responses above and beyond terminal training accuracy in the multiple linear regression analysis. The lack of a clear relation with intelligence might seem surprising given previous findings (see Section Introduction). One possible limitation of the current study is that we used the Raven Standard Progressive Matrices test, whereas in previous studies in which a correlation with fluid intelligence was observed, the Raven Advanced Progressive Matrices test was used (Winman et al., 2005; McDaniel et al., 2014; Little and McDaniel, 2015); the latter might be more appropriate in a sample mainly consisting of university students. However, evidence from previous studies does not in fact yield a very clear picture. The relation between intelligence and generalization style observed in function-learning and categorization tasks might have been inflated due to the complexity of the tasks employed. Even so, McDaniel et al. (2014) observed a correlation between intelligence and generalization style in one of three function-learning tasks only (*N*_1_ = 62, *N*_2_ = 76, *N*_3_ = 37). When the data of the three experiments were pooled, a weak overall correlation (*r* = 0.23) was obtained. From the work of Winman and colleagues it is unclear whether we should expect a correlation between intelligence and terminal accuracy or between intelligence and generalization style.

Second, we did not observe a correlation between SQ-R and generalization strategy, suggesting that systemizing was not related to rule use in the present patterning task. However, it has been argued that SQ-R is not be a valid instrument to measure systemizing (Ling et al., 2009; Morsanyi et al., 2012). Other researchers have even questioned whether systemizing is a meaningful concept to begin with (Ling et al., 2009; Morsanyi et al., 2012). The results reported here further fuel those debates.

Lastly, and most importantly, we investigated whether the tendency to focus on local details or global aspects of stimuli would influence the learning and generalization of patterning rules. Byrom and Murphy (2014) observed a correlation between global vs. local processing and accuracy of learning an A+/BC+/ABC− discrimination. Based on this observation, they suggested that, because negative patterning requires learning about configurations of stimuli (Byrom, 2013), a global processing advantage facilitates configural learning (Byrom and Murphy, 2014). This boils down to the assumption that how information is visually processed (i.e., whether the separate components rather than the configuration of the different parts are processed) influences the kind of associations that will be formed (i.e., whether associations between individual elements and the outcome will be formed as is the case in an elemental strategy or between configurations of elements and the outcome as is the case in a configural strategy). In other words, their assumption entails that visual processing style corresponds to mental representation style. Learning about positive and negative patterning problems simultaneously, as the participants in the current experiment were required to do, should also be enhanced by a configural strategy (and thus, according to the preceding analysis, by a global processing style). However, despite an almost identical Navon procedure (be it with a somewhat larger visual angle for stimulus presentation) and sufficient power, we did not observe a correlation between visual processing style and discrimination performance. We believe that a crucial difference between both experiments was the kind of patterning task employed. In the experiment of Byrom and Murphy each stimulus consisted out of nine colored shapes of one type (triangle, square, circle…) presented in a black grid of 6 × 6 squares. A compound of two stimuli consisted of the same grid, but filled with 18 shapes. Empty squares in the grid were filled with darkly colored circles. When focusing on the global aspects of a stimulus the difference between, say, a BC trial and an ABC trials was probably immediately apparent in that particular procedure. However, when focusing on the local aspects of the compounds those differences might not have been so clear, because the compounds shared a lot of local elements. Thus, people with a more global visual processing style might simply have been better in discriminating the different trial types of the negative patterning problem in the task used by Byrom and Murphy. Because there was no visual overlap between the different stimuli used in their linear discrimination control problem (D−/EF−/GHI+), visual processing type would not have influenced performance on that discrimination. We therefore argue that the correlation between visual processing style and association-formation performance observed by Byrom and Murphy was due to their specific experimental task and design. Importantly, we believe that there is no compelling evidence to extrapolate from their findings that configurations in a spatial sense reflect configurations in terms of mental representations. Of course, to ascertain that the nature of the stimuli used in the patterning task is indeed of crucial importance, it would be necessary to conduct a follow-up study in which all participants receive the same Navon task, but a patterning task that employs either the stimuli used by Byrom and Murphy (2014) or those used in the current experiment. If our analysis is correct, a correlation of visual processing style should be expected with performance in the former but not the latter patterning task.

In sum, our findings suggest that differences in learning speed and accuracy between participants are not sufficient to explain differences in the acquisition and generalization of associative patterning. While the relation of individual differences in intelligence with patterning performance remains unclear on the basis of the present results, individual differences in systemizing appear to be irrelevant for associative patterning. Finally, and most importantly, our results do not support previous suggestions of a principled association between the learning of patterning discriminations and visual processing style.

Further research will be needed to ascertain the exact source of previous observations of an apparent relation between visual processing style and patterning in associative learning. It would also be interesting to further investigate the acquisition pattern in more detail and whether rule-based and non-rule-based generalizers differ in their acquisition pattern. Our results seem to suggest that there is no difference in acquisition pattern between rule-based and non-rule-based generalizers. However, it is possible that more sensitive experiments would be able to uncover potential differences. Finally, to the best of our knowledge, no research has been conducted to separate the influence of the ability to abstract the rule of opposites from the tendency to apply this rule to a new set of stimuli on generalization performance.

## Author contributions

EM: Conception of the work, design of the study, data analysis and interpretation, writing, and revising the paper. EV: Conducted experiment, checking matlab scripts, data interpretation. JD: Conception of the work, revision of the paper. RD: Conception of the work, revision of the paper. TB: Conception of the work, design of the study, data interpretation, writing, and revising the paper.

### Conflict of interest statement

The authors declare that the research was conducted in the absence of any commercial or financial relationships that could be construed as a potential conflict of interest.

## Footnotes

^1^Because the criterion for determining outliers employed by Byrom and Murphy was rather strict, we also analyzed our data using a less strict criterion, by which only values that deviated more than 2.5 SD from the mean were removed. The same conclusions were reached when using this criterion.

^2^Byrom and Murphy also calculated a discrimination difference score, but over four blocks of training.

^3^Note that, according to Bayesian statistics, there is not indicating that the number of training blocks decreased with increasing RSPM scores for rule-based generalizers. It does, however, provide evidence for the absence of a correlation between those variables for non-rule-based generalizers.

## Appendix

**Table A1:** Correlations between patterning parameters on the one hand and RSPM and SQ-R on the other hand.

**Patterning parameter**	**Sub-group**		**RSPM**	**SQ-R**
Learning speed	Rule-based	*r*	−0.36	−0.04
		*p*	0.03	0.80
		95% *CI*	[−0.62, −0.04]	[−0.36,0.29]
		*BF*_*10*_	2.14	0.21
Overall accuracy	Rule-based	*r*	0.16	−0.02
		*p*	0.33	0.93
		95% *CI*	[−0.17, 0.46]	[−0.34, 0.31]
		*BF*_*10*_	0.32	0.21
Discrimination difference	Rule-based	*r*	0.18	−0.08
		*p*	0.29	0.63
		95% *CI*	[−0.15, 0.48]	[−0.40, 0.25]
		*BF*_*10*_	0.36	0.23
Learning speed	Feature-based	*r*	0.15	−0.06
		*p*	0.51	0.78
		95% *CI*	[−0.28, 0.53]	[−0.46, 0.36]
		*BF*_*10*_	0.32	0.27
Overall accuracy	Feature-based	*r*	0.16	−0.27
		*p*	0.47	0.21
		95% *CI*	[−0.27, 0.54]	[−0.62, 0.16]
		*BF*_*10*_	0.33	0.55
Discrimination difference	Feature-based	*r*	−0.001	0.16
		*p*	0.996	0.46
		95% *CI*	[−0.41, 0.41]	[−0.27, 0.54]
		*BF*_*10*_	0.26	0.34
Learning speed	All	*r*	−0.18	−0.07
		*p*	0.18	0.61
		95% *CI*	[−0.41, 0.08]	[−0.32, 0.19]
		*BF*_*10*_	0.39	0.18
Overall accuracy	All	*r*	0.19	−0.09
		*p*	0.14	0.50
		95% *CI*	[−0.06, 0.43]	[−0.34, 0.17]
		*BF*_*10*_	0.47	0.20
Discrimination difference	All	*r*	0.14	0.01
		*p*	0.30	0.93
		95% *CI*	[−0.12, 0.38]	[−0.24, 0.27]
		*BF*_*10*_	0.28	0.16
Percentage of rule-based generalization responses	All	*r*	0.25	0.06
		*p*	0.05	0.67
		95% *CI*	[−0.00, 0.48]	[−0.20, 0.31]
		*BF*_*10*_	1.05	0.18

*RSPM, Raven Standard Progressive Matrices*.
